# Characteristics of the Protoporphyrin IX Binding Sites on Human Serum Albumin Using Molecular Docking

**DOI:** 10.3390/molecules21111519

**Published:** 2016-11-17

**Authors:** Leszek Sułkowski, Bartosz Pawełczak, Mariola Chudzik, Małgorzata Maciążek-Jurczyk

**Affiliations:** 1 Department of General and Vascular Surgery, Regional Specialist Hospital, Bialska 104/118, 42-218 Częstochowa, Poland; 2 Department of Physical Pharmacy, School of Pharmacy with the Division of Laboratory Medicine in Sosnowiec, Medical University of Silesia, Jagiellońska 4, 41-200 Sosnowiec, Poland; bpawelczak@sum.edu.pl (B.P.); mchudzik@sum.edu.pl (M.C.); mmaciazek@sum.edu.pl (M.M.-J.)

**Keywords:** photodynamic diagnosis, photodynamic therapy, protoporphyrin IX, human serum albumin, UV-VIS, emission fluorescence, molecular docking

## Abstract

Human serum albumin (HSA) is the main plasma protein responsible for a distribution of drugs in the human circulatory system. The binding to HSA is one of the factors that determines both the pharmacological actions and the side effects of drugs. The derivative of heme, protoporphyrin IX (PpIX), is a hydrophobic photosensitizer widely used in photodynamic diagnosis and therapy of various malignant disorders. Using absorption and fluorescence spectroscopy, it has been demonstrated that PpIX forms complexes with HSA. Its binding sites in the tertiary structure of HSA were found in the subdomains IB and IIA. PpIX binds to HSA in one class of binding sites with the association constant of 1.68 × 10^5^ M^−1^ and 2.30 × 10^5^ M^−1^ for an excitation at wavelength λ_ex_ = 280 nm and 295 nm, respectively. The binding interactions between HSA and PpIX have been studied by means of molecular docking simulation using the CLC Drug Discovery Workbench (CLC DDWB) computer program. PpIX creates a strong ‘sandwich-type’ complex between its highly conjugated porphine system and aromatic side chains of tryptophan and tyrosine. In summary, fluorescent studies on binding interactions between HSA and PpIX have been confirmed by the results of computer simulation.

## 1. Introduction

The laser light affecting tissues can be used in the treatment of cancers and pre-cancerous lesions. An illumination initiates a photochemical reaction leading to the formation of cytotoxic compounds, which induce cell death [1,2]. Damage of the mitochondrial membrane, liposomes, Golgi apparatus, cytoplasmatic reticulum and nuclear membrane has been observed. This reaction cannot occur without a photosensitizer [3,4]. Photodynamic diagnosis (PDD) and therapy (PDT) take advantage of this phenomenon. The prevalence of the photodynamic reaction in neoplasm tissue and its surroundings is essential.

PDD and PDT of cancer and pre-cancerous lesions require photosensitizers, which are activated by light [1,4]. Any particular photosensitizer cannot be highly effective in the treatment of different neoplasms. Its effectiveness depends on the chemical structure, lipo- or hydro-phobic properties, carrier and solvent, type of neoplasm, pH of intracellular and extracellular fluid, concentration of photosensitizer and the type of complexes formed with carriers.

The porphyrin is a macrocycle composed of four pyrrole rings bridged by four sp2 hybridized carbon atoms [2,5]. Protoporphyrin IX (PpIX) is a clinically useful hydrophobic photosensitizer, which accumulates the lipid structures of cancer cells. PpIX is a heme metabolite [1,6,7]. PpIX is used in both PDD and PDT.

Since human serum albumin (HSA) is a component of the circulatory system [8], the understanding of porphyrin binding to HSA is crucial for PDD and PDT success [5,9,10].

One of the most important properties of HSA is its ability to bind and transport many endo- and exo-genic compounds, which have no designed transport proteins. Nevertheless, compounds having specific transport proteins can also be transported by HSA. The formation of albumin-ligand complexes in the blood serum preserves ligands against oxidation, reduces their toxicity and improves their solubility, and therefore, the formation of the complexes improves the transportation of ligands.

Preclinical studies are crucial in order to support and develop PDD and PDT. In our study, absorption and fluorescence UV spectroscopy was used to find PpIX binding sites in transporting protein tertiary structure. Molecular docking was applied to check and confirm our experimental study. The CLC Drug Discovery Workbench (CLC DDWB) molecular docking computer program is an integrated virtual environment for exploring various biochemical processes, such as binding of small molecules (medicines, hormones, vitamins or minerals) to bioactive macromolecules (carrier proteins, receptors or nucleic acids). In the study, molecular docking simulation was used for the simulation of binding interactions between the macromolecule of HSA and the molecule of PpIX.

## 2. Results and Discussion

### 2.1. Formation of the PpIX-HSA Complex

**Figure 1:**
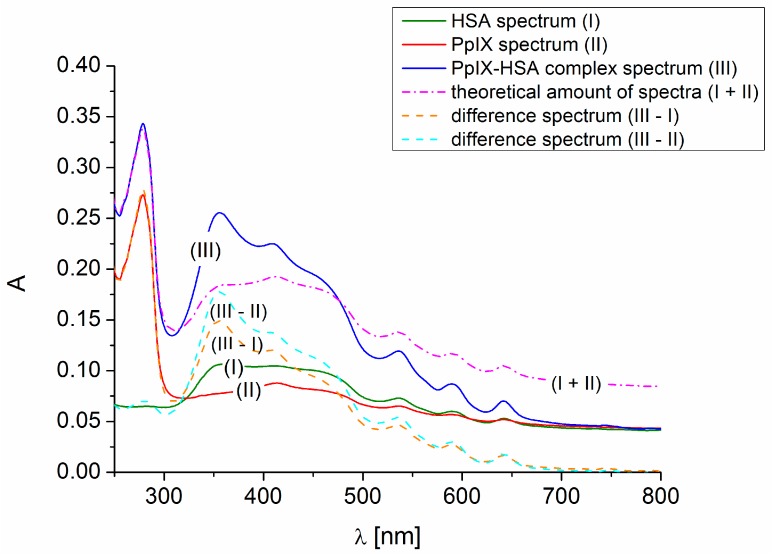
Difference spectra of protoporphyrin IX (PpIX) (5 × 10^−6^ M)-human serum albumin (HSA) (1 × 10^−6^ M) systems.

The absorption spectra of HSA (I), PpIX (II), as well as the PpIX-HSA system (III) are shown in Figure 1. Difference spectra (III − I and III − II) were also recorded. The absorption spectrum (I + II) that arose by the addition of spectra of PpIX and HSA does not overlap the spectrum of the complex (III). Similarly, difference spectra (III − I) and (III − II) do not overlap spectra (II) and (I), respectively. A new quality in the PpIX-HSA system appeared. This suggests that a complex between HSA and PpIX may be formed.

To obtain evidence of the complex formation between HSA and PpIX, fluorescence analysis was conducted.

**Figure 2:**
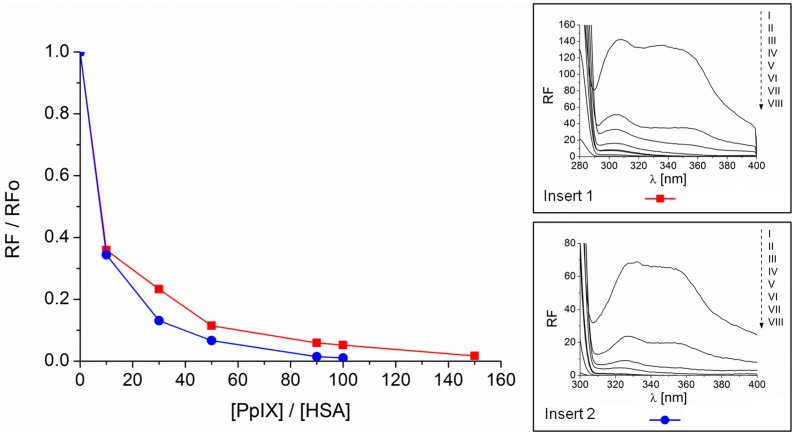
Quenching of HSA fluorescence by PpIX obtained for 280 nm (${}_{▬■▬}$) and 295 nm (${}_{▬●▬}$) excitation wavelength. In the inserts: fluorescence emission spectra of HSA at concentration 1 × 10^−6^ M (I) and HSA in the presence of increasing PpIX concentration in range from 1 × 10^−5^ M to 3 × 10^−4^ M (II, III, IV, V, VI, VII, VIII) excited at 280 nm (Insert 1) and 295 nm (Insert 2).

The fluorescence of HSA excited at both wavelength λ_ex_ = 280 nm and 295 nm was quenched by PpIX (Figure 2). The fluorescence of HSA excited at both λ_ex_ = 280 nm and 295 nm decreased in the presence of PpIX (Figure 2). A hypsochromic shift of the HSA maximum emission fluorescence at λ_ex_ = 334 and 340 nm by 7 and 5 nm, respectively, is observed. The quenching effect of PpIX on the fluorescence of HSA increases with increasing concentration of the ligand. The quenching of the protein fluorescence may be an effect of energy transfer between tryptophan (Trp) and tyrosine (Tyr) fluorophores in the HSA and chromophores in the PpIX. That may occur when the donor-acceptor distance does not exceed 10 nm [11]. This confirms the formation of the complex PpIX-HSA. A blue-shift of the emission fluorescence maximum is caused by conformational changes in the HSA, which involve the indole ring located within the hydrophobic pocket in the serum albumin molecule. The environment of the fluorophore thus becomes less polar. The effect of solvent polarity on serum albumin fluorescence has been described previously [12]. The blue-shift of HSA emission fluorescence excited at λ_ex_ = 295 nm can be attributed to the significant alteration within the fluorophore’s environment, demonstrating the exposure of Trp214 to the solvent.

**Figure 3:**
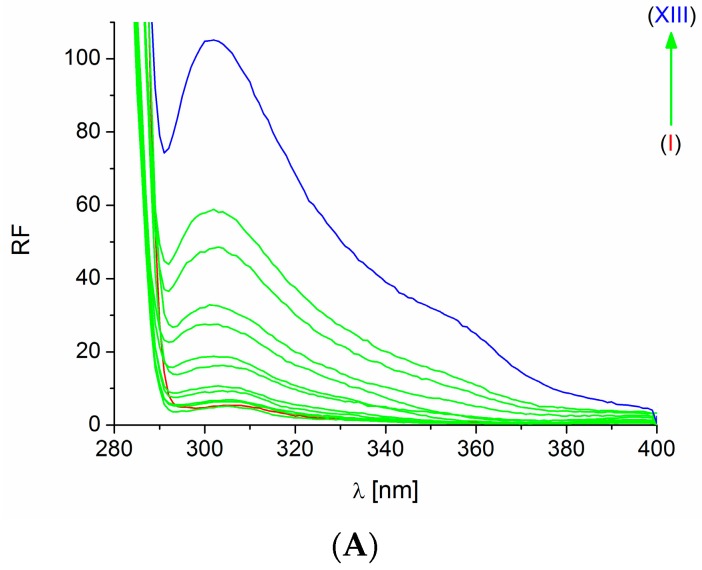

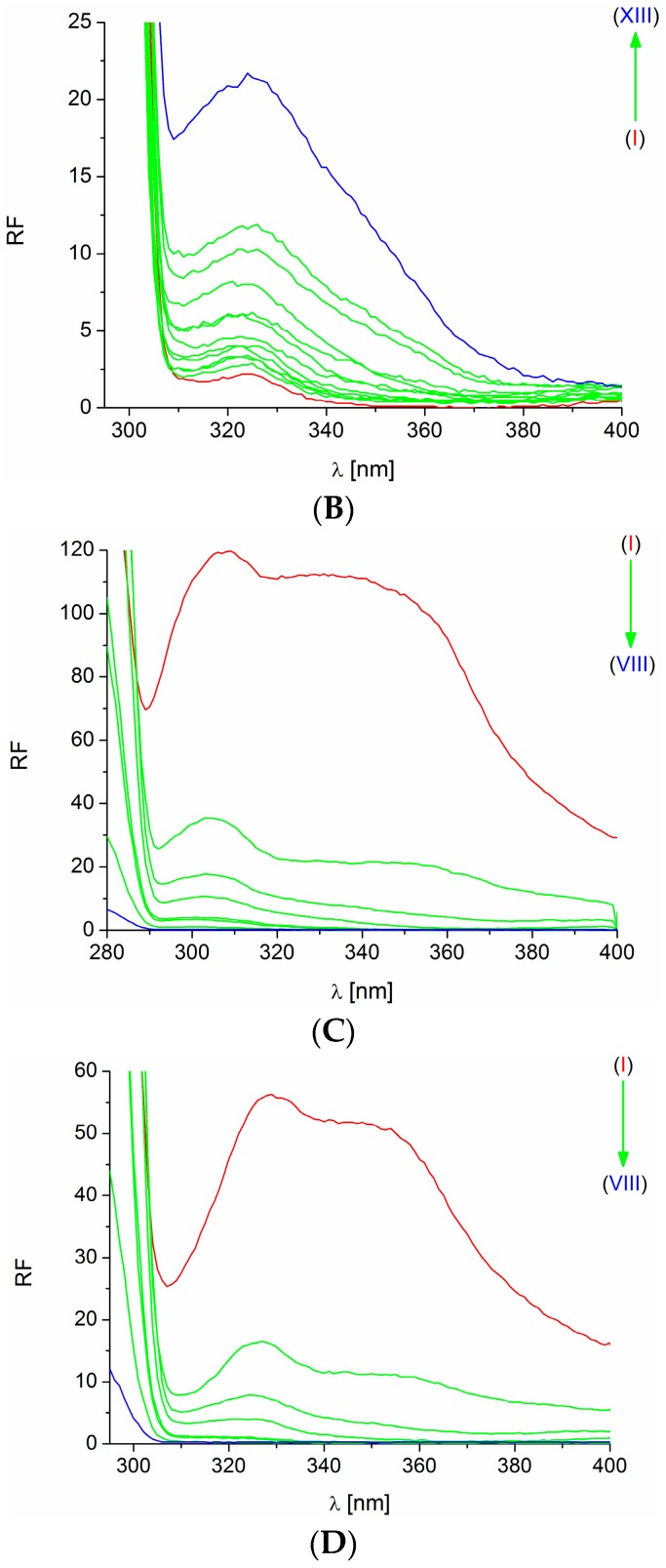
The increase of PpIX (I, 5 × 10^−5^ M) fluorescence excited at λ_ex_ = 280 nm (**A**) and 295 nm (**B**) in the presence of HSA (II, 2 × 10^−7^ M; III, 3 × 10^−7^ M; IV, 4 × 10^−7^ M; V, 5 × 10^−7^ M; VI, 7.5 × 10^−7^ M; VII, 1 × 10^−6^ M; VIII, 1.25 × 10^−6^ M; IX, 2 × 10^−6^ M; X, 2.5 × 10^−6^ M; XI, 4 × 10^−6^ M; XII, 5 × 10^−6^ M; XIII, 9 × 10^−6^ M). Quenching of HSA (I, 7.5 × 10^−7^ M) fluorescence excited at λ_ex_ = 280 nm (**C**) and 295 nm (**D**) in the presence of PpIX (II, 1 × 10^−5^ M; III, 3 × 10^−5^ M; IV, 5 × 10^−5^ M; V, 9 × 10^−5^ M; VI, 1 × 10^−4^ M; VII, 1.5 × 10^−4^ M; VIII, 3 × 10^−4^ M).

The presence of HSA causes the increase of PpIX fluorescence excited at both λ_ex_ = 280 nm and 295 nm (Figure 3).

Both phenomena, i.e., quenching of HSA fluorescence by PpIX (Figure 2 and Figure 3) and an increase of PpIX fluorescence in the presence of HSA (Figure 3), indicate the possibility of an interaction between HSA and PpIX in the binding sites where the Trp and Tyr residues are located, i.e., IB, IIA and IIIA [13,14].

### 2.2. Association and Stern–Volmer Constants

**Figure 4:**
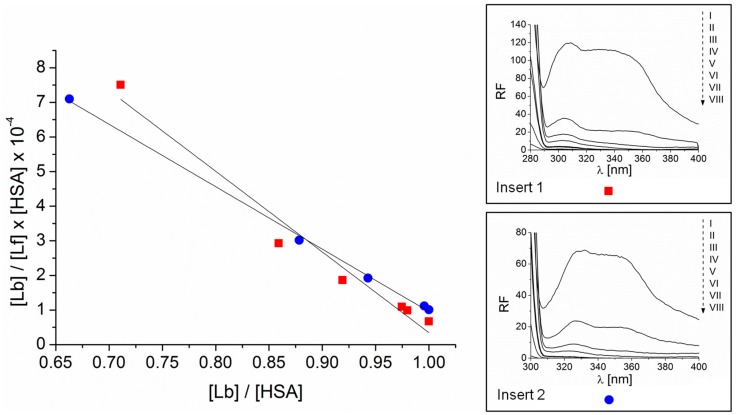
Scatchard dependence for the PpIX-HSA complex excited at 280 nm (${}_{■}$) (*y* = −23.317*x* + 23.663) and 295 nm (${}_{●}$) (*y* = −18.44*x* + 18.999). Data determined from emission fluorescence spectra. In the insert 1: fluorescence emission spectra of HSA at concentration 7.5 × 10^−7^ M (I) and HSA in the presence of increasing PpIX concentration in range from 1 × 10^−5^ M to 3 × 10^−4^ M (II, III, IV, V, VI, VII, VIII) excited at 280 nm. In the insert 2: fluorescence emission spectra of HSA at concentration 1 × 10^−6^ M (I) and HSA in the presence of increasing PpIX concentration in range from 1 × 10^−5^ M to 3 × 10^−4^ M (II, III, IV, V, VI, VII, VIII) excited at 295 nm. [Lb], bound ligand concentration; [Lf], free ligand concentration.

**Figure 5:**
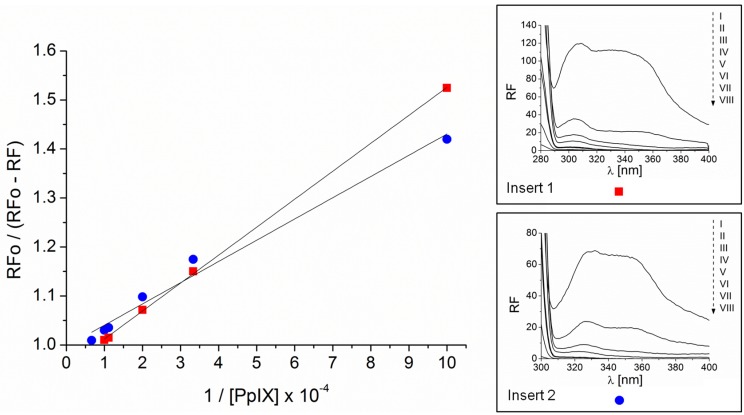
Stern-Volmer dependence for the PpIX-HSA complex excited at 280 nm (${}_{■}$) (*y* = 0.0434*x* + 0.9971) and 295 nm (${}_{●}$) (*y* = 0.0571*x* + 0.9551). Data determined from emission fluorescence spectra. In the insert 1: fluorescence emission spectra of HSA at concentration 7.5 × 10^−7^ M (I) and HSA in the presence of increasing PpIX concentration in range from 1 × 10^−5^ M to 3 × 10^−4^ M (II, III, IV, V, VI, VII, VIII) excited at 280 nm. In the insert 2: fluorescence emission spectra of HSA at concentration 1 × 10^−6^ M (I) and HSA in the presence of increasing PpIX concentration in range from 1 × 10^−5^ M to 3×10^−4^ M (II, III, IV, V, VI, VII, VIII) excited at 295 nm.

In order to estimate the stability and the strength of the binding of a photosensitizer with protein, the association (K_a_) and Stern–Volmer (K_S-V_) constants were determined (Figure 4 and Figure 5, respectively). The shape of the plot (Figure 4) points to the presence of one class of binding sites for PpIX molecules in the HSA tertiary structure. The Stern–Volmer constant allows estimating how close the protein fluorophore PpIX is located. Both K_a_ and K_S-V_ (Table 1) are of the same order. This may suggest that within one class of binding sites on the HSA tertiary structure, PpIX forms a stable complex in the subdomain IIA where Trp 214 is located and/or in other sites (IB, IIIA) containing Tyr residues.

Spatial structure of the PpIX molecule bound to HSA may have an effect on the strength of binding and the distance between the ligand and the fluorophore of the protein. π-π interactions leading to “sandwich-type” structures should be taken into account [15].

### 2.3. Molecular Properties of PpIX

**Figure 6:**
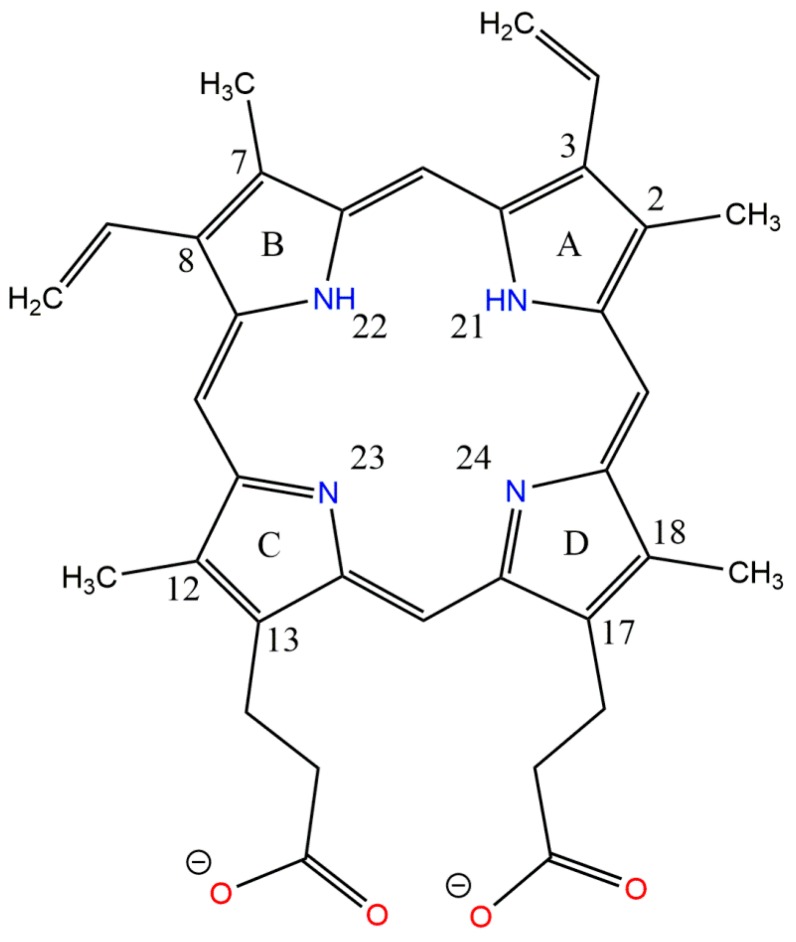
Chemical structure of the dominant PpIX tautomer in aqueous solution at pH 7.4. Atoms and rings are numbered in order of the docking results’ interpretation.

**Figure 7:**
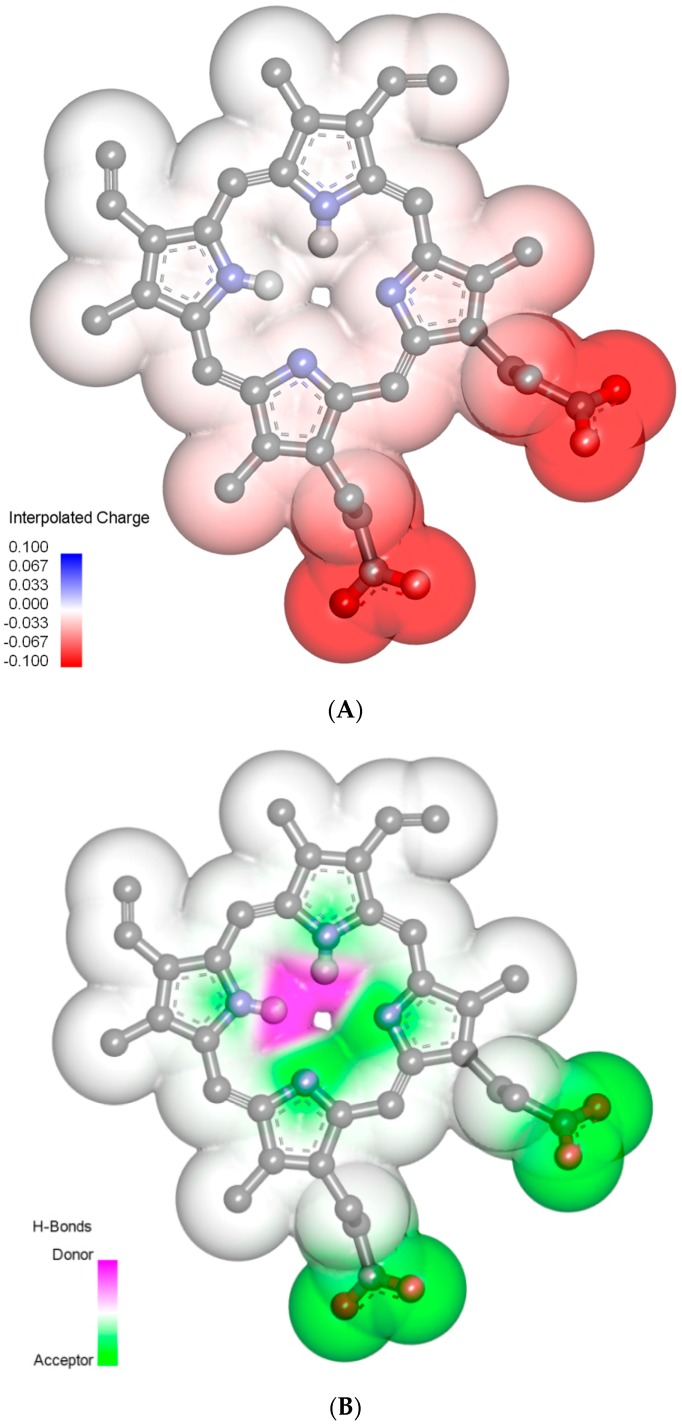
Three-dimensional structure of the PpIX molecule in the energy optimized conformation used in molecular docking. (**A**) Interpolated charges; (**B**) hydrogen bond donors and acceptors. Models are shown in the ball and stick representation with the van der Waals surface (atom surfaces are scaled to 100% of the van der Waals radii). For the sake of clarity, only polar hydrogen atoms are displayed.

The chemical 2D and 3D structure of PpIX with minimum energy conformation and its predicted physico-chemical properties are shown in Figure 6 and Figure 7. PpIX is a highly pharmacologically-active heterocyclic organic compound composed of four conjugated pyrrole rings (A, B, C and D) connected via four methane bridges. The macrocycle system of PpIX is substituted by four methyl groups at positions C(3), C(7), C(12), C(17), two vinyl groups at positions C(8), C(13) and two propionic acid groups at positions C(2), C(18). The pro-tropic tautomerism in macrocyclic porphine ring at positions N(21)H/NoH, N(22)H/NoH, N(23)H/NoH and N(24)H/NoH has been the subject of several studies [16,17,18,19]. Computer analysis of the PpIX structure suggested that this compound may exist in equilibrium as four tautomeric forms N(21)H-N(22)H ↔ N(22)H-N(23)H ↔ N(23)H-N(24)H ↔ N(22)H-N(24)H; however, in solutions at physiological pH, the major tautomer of PpIX is N(22)H-N(23)H (about 49% distribution) (Figure 6). PpIX is a weak acidic compound, which dissociates in solutions at pH 7.4. PpIX mainly occurs as an anionic form (about 99.65% in a mixture of neutral and ionic forms) (Figure 7A). Additionally, the PpIX molecule is distinguished by a high potential of the formation of hydrogen bonds and hydrophobic π-π stacking interactions (Figure 7B).

### 2.4. Visualization of the PpIX Binding Sites in the HSA Molecule

The HSA primary structure is a sequence of 585 amino acids, which form one polypeptide chain stabilized by 17 disulfide bridges [14]. The secondary structure of HSA based on the X-ray crystallographic analysis shows that the HSA polypeptide chain forms about thirty α-helices, many coiled-coils and none β-sheets [20]. The tertiary HSA structure consists of three homologous domains (I, II and III) formed as the heart-shaped conformation [21]. Each domain is comprised of two subdomains (A and B) [14]. Each subdomain A is composed of six (h1–h6) α-helices, while the subdomain B of four (h1–h4) α-helices [20]. HSA contains one tryptophan residue (Trp 214) located deeply in its hydrophobic matrix in Subdomain IIA [14]. Sudlow [13] classified two principal drug-binding sites in HSA molecule located in Subdomains IIA (Sudlow’s Site 1) and IIIA (Sudlow’s Site 2).

**Figure 8:**
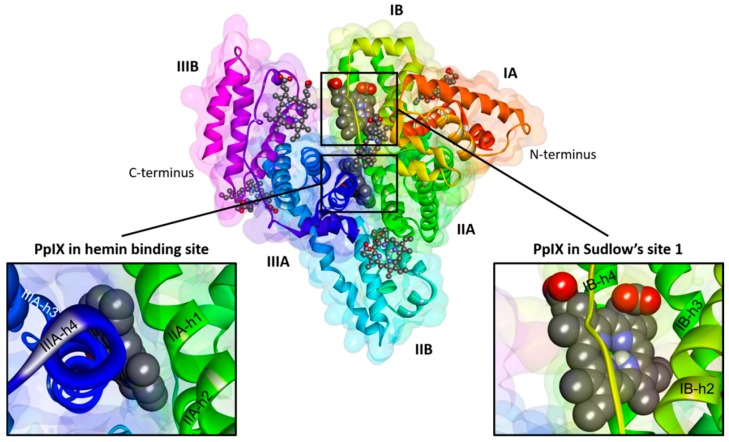
Macromolecule of HSA (PDB 1AO6) complexed with the PpIX molecules. The protein backbone is shown schematically as a ribbon. Each subdomain of HSA is marked according to the model proposed by Carter and Ho [14]. The best docking results (number 1 and 2) are shown in the CPK representation. Other docking results are shown in the ball and stick representation. For the sake of clarity, only polar hydrogen atoms are displayed.

**Figure 9:**
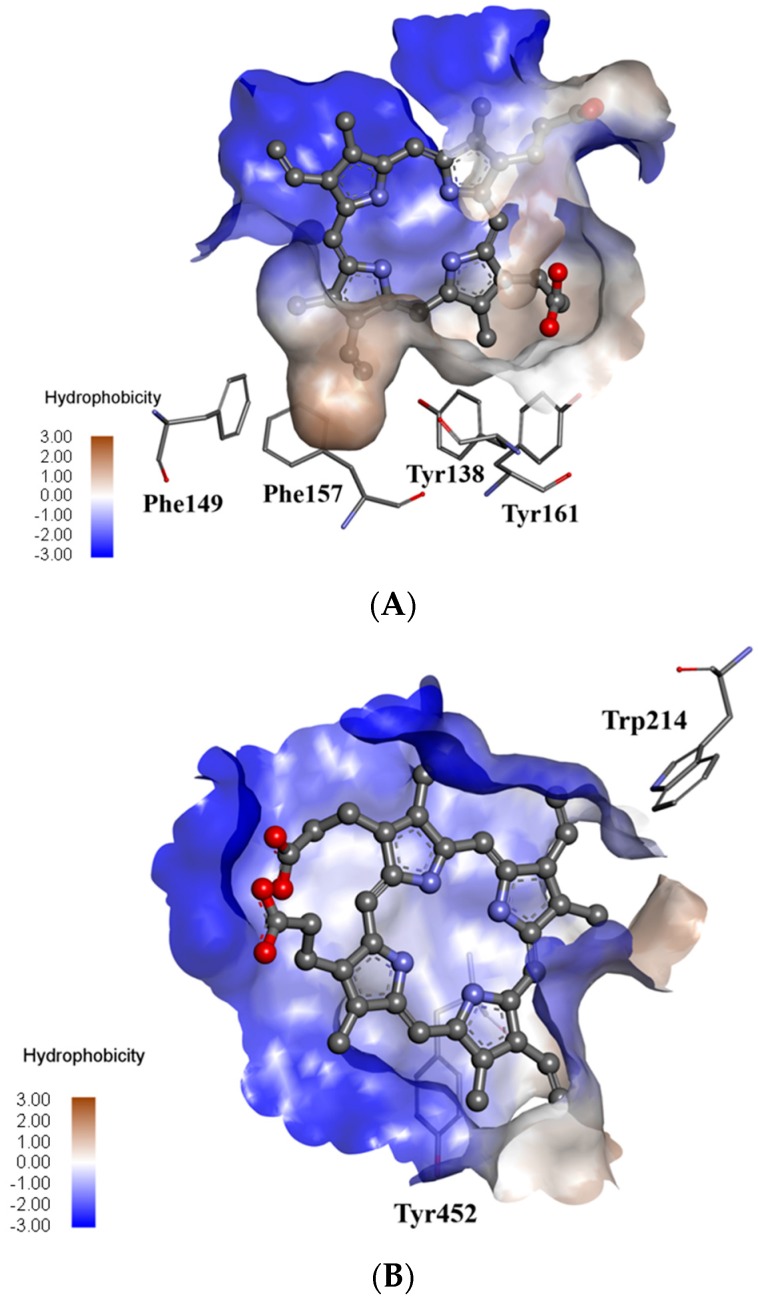
Superposition of the PpIX molecule inside the hemin site (**A**) and Sudlow’s Site 1 (**B**) of the HSA macromolecule. The geometry of the binding sites is colored according to its hydrophobicity. The ligand is shown in the ball and stick representation. Aromatic amino acids are shown in the thin stick representation. For the sake of clarity, only polar hydrogen atoms are displayed.

As an introduction to molecular modelling, seven possible binding sites, which represent the free space inside the HSA structure able to interact with various ligands, have been identified. The ligand binding sites of HSA detected in this study are shown in Figure 8. Among all of the detected ligand binding sites of has, the lowest interaction energy results for docked PpIX were found only for Site 1 (hemin site [22]) and Site 2 (Sudlow’s Site 1 [13]) (Figure 8). These sites were selected as proposed binding sites for PpIX. The localization and detailed characterization of the detected ligand binding sites and the energy of their interactions with PpIX are presented in Table 2. The first site (Site 1) is the largest one and located deeply in the hydrophobic matrix of Subdomain IB (Figure 9A).The second site is found deeply in the hydrophobic pocket of Subdomain IIA inside the protein core (Figure 9B).

### 2.5. Binding Site Interactions between PpIX and HSA Amino Acids

**Figure 10:**
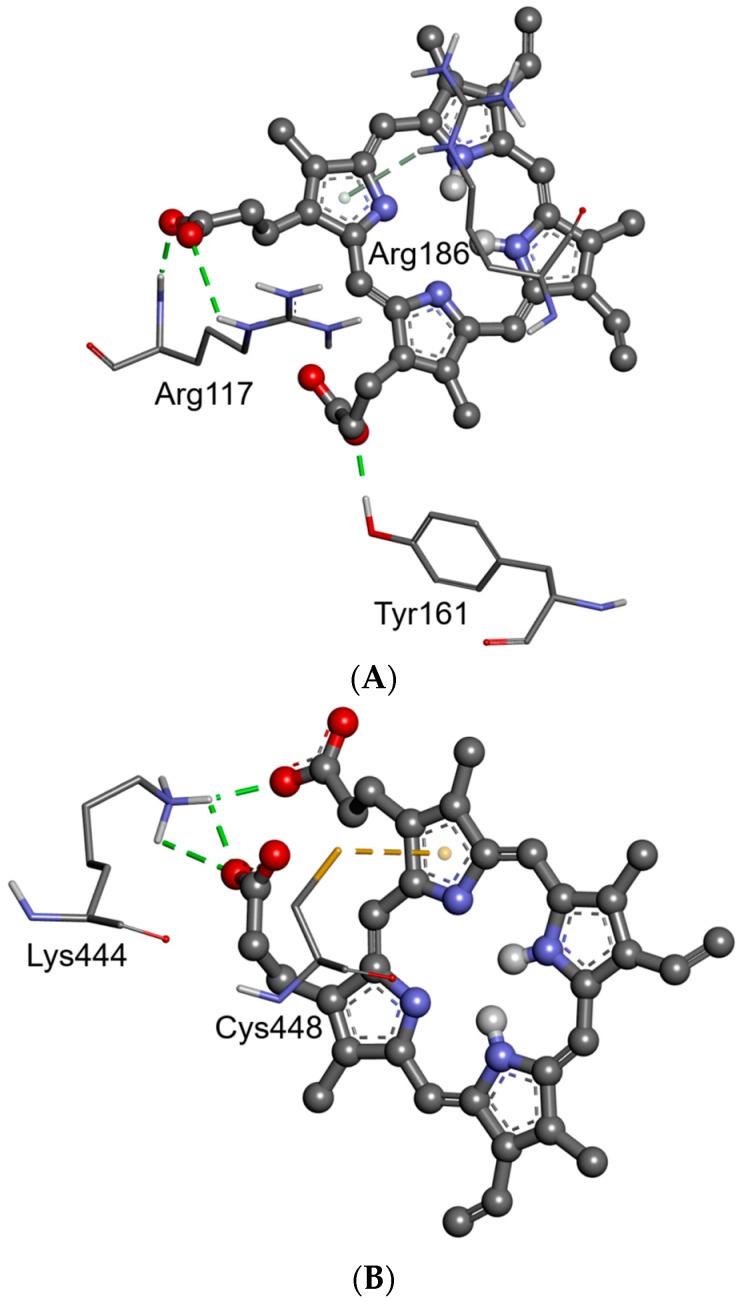
Hydrogen bonds and miscellaneous (sulfur) interactions between the residues of HSA and the molecules of PpIX in the hemin site (**A**) and Sudlow’s Site 1 (**B**). Interactions between ligand and amino acids are visualized in dashed lines and colored according to their type (conventional and non-classical H-bonds are green and olive; the miscellaneous interaction is yellow). Ligands and are shown in the ball and stick representation, respectively.

**Figure 11:**
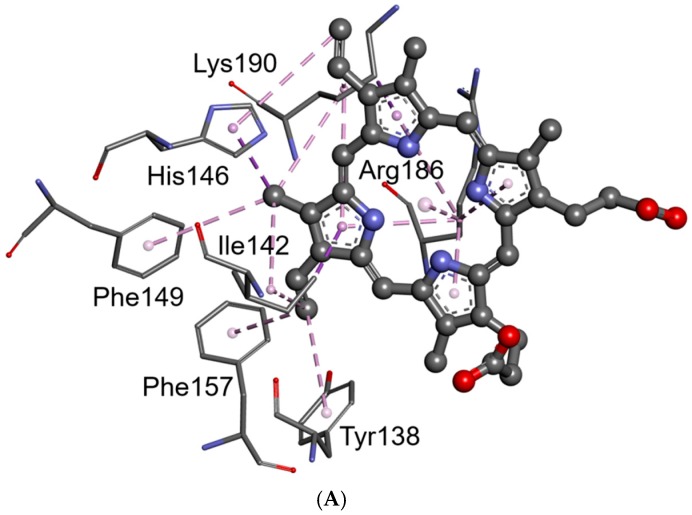

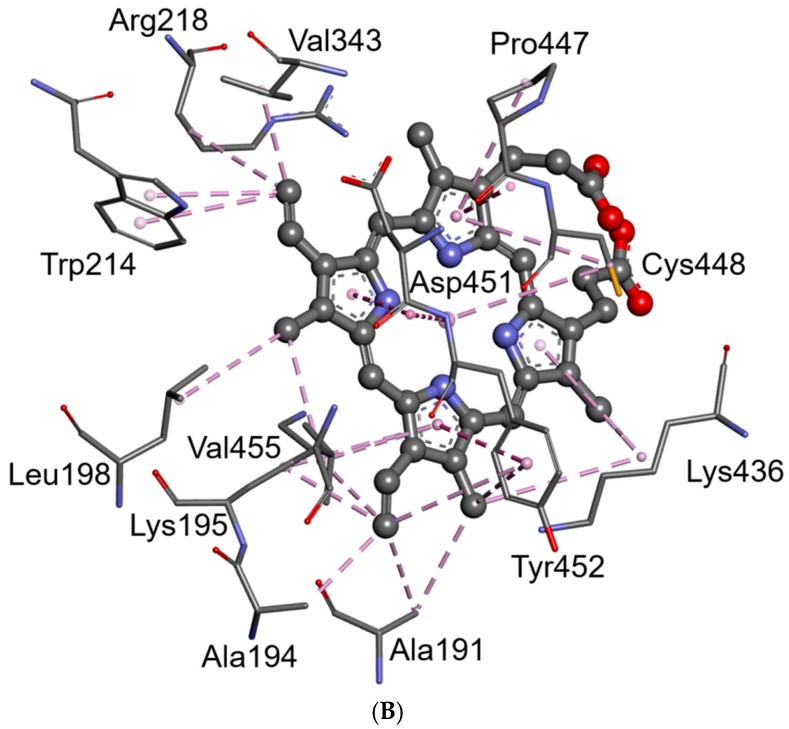
Hydrophobic interactions between the molecules of PpIX and the residues of HSA around the hemin site (**A**) and Sudlow’s Site 1 (**B**). Ligands and residues are shown in the ball and stick and thin stick representation, respectively.

The results of computer simulation showed that there are many common interaction residues submitted for the binding of PpIX into Sites 1 and 2 (Figure 10 and Figure 11, Table 3). There was a combined hydrogen bonding and hydrophobic interaction pattern observed upon docking of the PpIX into Sites 1 and 2 of HSA. The docked ligand manifested multicenter hydrophobic interaction between the highly conjugated system of PpIX and the side chains of amino acids located inside Site 1 (Tyr 138, Ile 142, His 146, Phe 149, Phe 157, Arg 186 and Lys 190) and Site 2 (Ala 191, Ala 194, Lys 195, Leu 198, Trp 214, Arg 218, Val 343, Lys 436, Lys 444, Pro 447, Cys 448, Asp 451, Tyr 452 and Val 455) (Figure 10, Table 3). PpIX-HSA complexes are stabilized by π-π stacking interactions with aromatic amino acids into Sites 1 (Tyr 138, Phe 149, Phe 157) and 2 (Trp 214, Tyr 452). Furthermore, hydrogen bonding interactions play an important role in the binding of PpIX. The most important amino acid residues involved in hydrogen bonding interactions are Arg117 (Site 1), Tyr 161 (Site 1) and Lys 444 (Site 2). In summary, these residues that show close interactions with the PpIX molecule inside Site 1 and Site 2 may appear to be the key contributors to the high binding affinity. Even though internal stabilization by intramolecular hydrogen bonds was observed in the ligand structure after docking simulation for both binding Sites 1 and 2, the docking results suggest that PpIX has a good binding affinity for the HSA.

**Figure 12:**
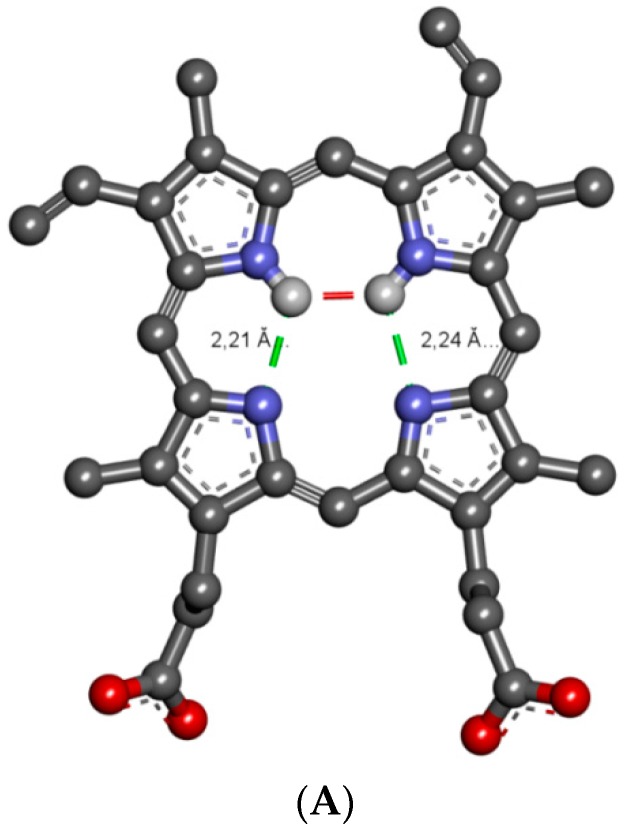

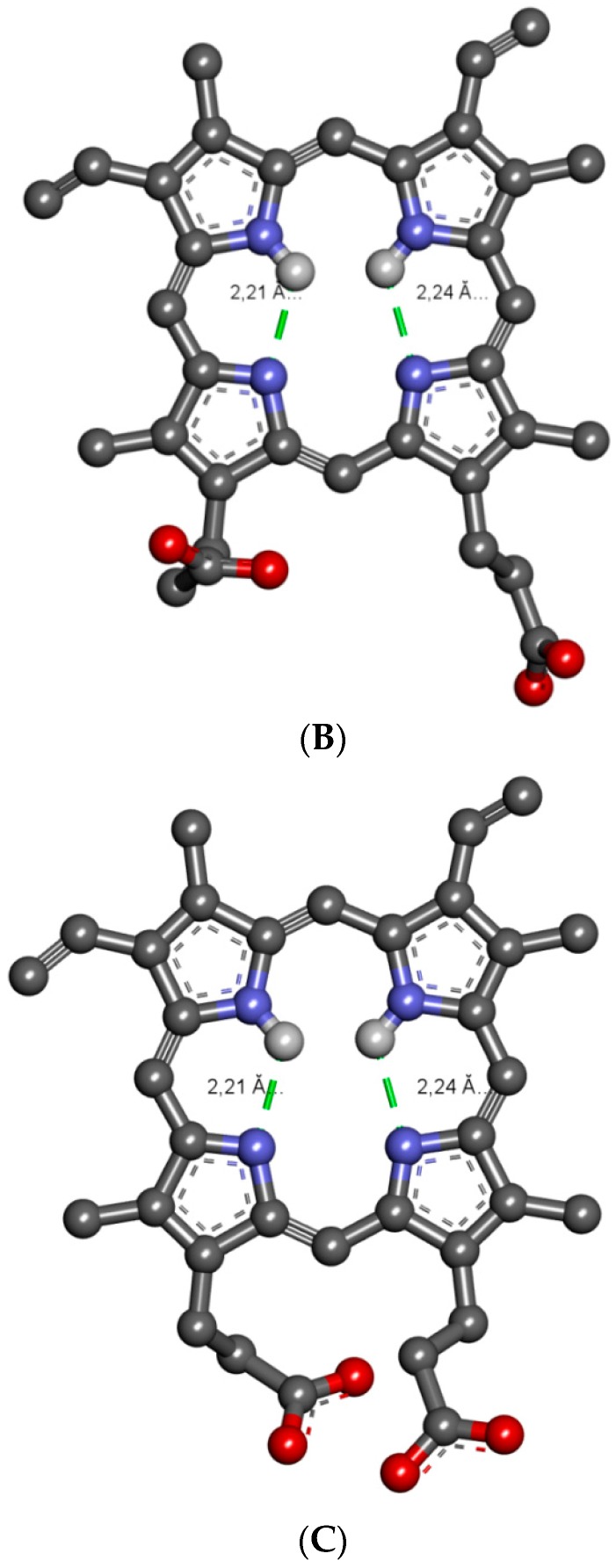
The differences in molecular conformations of PpIX before and after computational simulation. (**A**) Initial conformation of PpIX; (**B**) conformation of PpIX bound in the hemin site; (**C**) conformation of PpIX bound in Sudlow’s Site 1. Molecules are shown in the ball and stick representation of their aromatic forms. Intramolecular hydrogen bonds are highlighted with dashed lines (1 Å = 0.1 nm). For the sake of clarity, only polar hydrogen atoms are displayed.

As a result of the interactions of the PpIX molecule with the HSA, amino acid residue changes in the spatial conformation of the ligand appear. The comparison of the molecular PpIX structure before molecular docking with that isolated from the PpIX-HSA complex after docking is shown in Figure 12. The changes in the PpIX spatial structure concern setting in 3D the vinyl group in C(3) and C(8) positions, as well as propionic acid residues in C(13) and C(17). Due to the formation of strong hydrogen bonds with Arg 117 and Tyr 161 (Site 1) and Lys 444 (Site 2), the most significant changes in PpIX conformation are observed.

## 3. Materials and Methods

### 3.1. Chemicals

HSA, Fraction V, crystallized and lyophilized, was obtained from ICN Biomedicals Inc., Aurora, OH, USA.

PpIX (3,7,12,17-tetramethyl-8,13-diwvnyl-2,18-porfinodipropionic acid) was obtained from Aldrich Chem. Co., Milwaukee, WI, USA.

### 3.2. Absorption UV-VIS Analysis

The UV-VIS spectra were recorded on a spectrophotometer (Jasco International Co., Ltd., Tokyo, Japan), the Jasco V-530. The wavelength accuracy was ±1 nm; the photometric accuracy was ±0.002 Abs. at 0.5 Abs. All PpIX and HSA samples were prepared in sodium phosphate buffer (pH 7.4).

The UV-VIS spectra of the PpIX-HSA system were recorded for two systems: the first containing a constant PpIX concentration and the second containing a constant HSA concentration. The differences of the UV-VIS spectra of the PpIX-HSA system were recorded, as well. The intensity and shape of all of the recorded spectra were corrected for the absorption of the buffer using software (Spectra Manager version 1.55.00, Jasco International Co., Ltd., Tokyo, Japan) supplied by Jasco.

### 3.3. Fluorescence Analysis

Emission fluorescence spectra were recorded with 1 cm × 1 cm × 4 cm quartz cells on a KONTRON SFM-25 Instrument AG (Kontron AG, Zurich, Switzerland), 30 min after sample preparation at 25 °C. Correcting the error of the apparatus for wavelength and relative fluorescence was equal to λ = ±1 nm, RF = ±0.01, respectively. For excitation wavelength λ_ex_ = 280 nm, 295 nm and 405 nm, the scan range was 280–400 nm, 295–400 nm and 550–750 nm, respectively.

By using fluorescence data, the quenching curves, i.e., (*f*([PPIX]:[HSA]) = F/F_0_), of HSA in the presence of PpIX have been plotted (F_0_, fluorescence of HSA in the absence of PpIX; F, fluorescence of HSA in the presence of PpIX). The quenching of HSA fluorescence caused by PpIX was used to determine [PpIX_b_] from:

$$\left[{\mathrm{PpIX}}_{b}\right]=\;\frac{\Delta F}{\Delta F_{\max}}\cdot {\left[\mathrm{HSA}\right]}_{t}$$

where $\Delta F=F_{0}-F$; [HSA]_t_ the total concentration of HSA; $\Delta F_{\max}$ the maximal fluorescence decrease (to determine that the $\Delta F_{\max}$ afixed concentration of HSA was titrated with increasing concentration of PpIX); $\frac{1}{\Delta F_{\max}}$ is an intersect of the ordinate axis on the plot $\frac{1}{\Delta F}$ versus $\frac{1}{\left[{\mathrm{PpIX}}_{\mathrm{total}}\right]}$.

The concentration of unbound (free) PpIX was obtained from:

$$\left[PpIX_{f}\right]=\left[PpIX_{total}\right]-\left[PpIX_{b}\right]$$

### 3.4. Association Constants and Quenching Constants

The association constants of PpIX to HSA were estimated by the method described by Hiratsuka [25].

The Stern–Volmer constants were calculated according to the equation [11]:

RFo/RF = 1 + K_S-V_ × [Q]

where RFo and RF are the fluorescence intensities in the absence and presence of the quencher, respectively; K_S-V_ is the collisional quenching constant; [Q] is the quencher (PpIX) in the complex HSA-PpIX.

### 3.5. Computational Simulation

The docking experiment was performed using the CLC Drug Discovery Workbench (CLC DDWB) computer program [23]. Docking results were graphically elaborated using the BIOVIA Discovery Studio Visualizer (DS) software [24].

The crystal structure of HSA (Chains A and B) required for computer simulation was downloaded from the Protein Data Bank (PDB) database [26] with 4-letter PDB accession 1AO6 and imported to the CLC DDWB computer program. The downloaded protein structure was obtained by the X-ray diffraction method with a resolution of 2.5 Å by Sugio et al. [20]. The loaded file 1AO6.pdb contains two single, unglycosylated and ligand-free polypeptide chains, A and B. The chain A was chosen to be a target protein structure. Before the docking experiment, the chain B and all crystallographic water molecules were removed from protein file 1AO6.pdb. Then, the ionizable amino acid residues of the protein’s Chain A were set to their protonation states, which exist in physiological conditions at pH 7.4. Amino acids with positive electrically-charged side chains such as Arg (PDB atom: NH1, NH2 and NE), His (PDB atom: ND1) and Lys (PDB atom: NZ) were protonated, while those of amino acids with negative electrically-charged side chains, such as Asp (PDB atom: OD1 and OD2) and Glu (PDB atom: OE1 and OE2), were deprotonated.

The two-dimensional (2D) structure of PpIX was built using the ChemDraw Ultra [27] program and converted to the three-dimensional (3D) representation by the use of the Chem3D Ultra [27] program. Subsequently, the overall geometry optimizations and partial atomic charge distribution calculations of the ligands were performed with the same program using the Austin Model 1 (AM1) semi-empirical molecular orbital method [28]. The CLC DDWB program and Marvin Sketch [29] software were used for characterizing the molecular properties of PpIX, including molecular mass, number of rotatable bonds, XlogP, hydrogen bond acceptors and donors, van der Waals surface, water solubility, dissociation constant (pK), major microspecies and major tautomeric forms at pH 7.4.

### 3.6. Molecular Docking Procedure

Molecular docking simulation consists of four steps: finding binding sites, docking ligand to the binding sites, virtual screening and, finally, visualization of the docking results.

Initially, potential ligand-binding sites in the HSA structure were automatically detected by the Find Binding Pockets algorithm implemented in CLC DDWB. Then, the detected binding sites were classified according to their molar volumes (all sites with a volume less than 50 Å^3^ were discarded) and analyzed by selecting neighboring amino acids within a 10–15 Å radius from the center of each binding site. In the second step, the PpIX molecule was fitted separately into each detected ligand-binding site of HSA by the CLC DDWB search algorithm with standard docking simulation settings (No. of iterations for each ligand = 100; number of docking results returned for each ligand = 1). In this step, the rotation around flexible bonds was changing the conformation of the ligand. During the experiment, the binding energy for each obtained complex was automatically scored by the PLANTS_PLP_ scoring function used in the CLC DDWB program and presented in arbitrary units (a.u.) [30]. Finally, all solutions of protein-ligand complexes were ranked according to their docked energy (virtual screening). A very negative score corresponds to a strong binding, while a less negative or even a positive score corresponds to a weak or non-existing binding. In the last step, the complexes with the best score were visualized as the final docking results.

## 4. Conclusions

The binding of medicines to plasma proteins determines their pharmacological actions and side effects. A significant role in the drug carriage is played by HSA. In this work, spectroscopic and molecular docking studies were applied in order to identify the likely PpIX binding sites of HSA and to explain the mechanism of binding the PpIX to this carrier protein.

The formation of the complex between PpIX and HSA was confirmed by the analysis of the difference of the UV-VIS spectra and spectrofluorescence quenching analysis.

Based on the absorption analysis, it was concluded that PpIX may form complexes with HSA in human plasma. Additionally, the fluorescence analysis results show that PpIX is able to interact with both Trp and Tyr residues. These results suggest that PpIX most likely binds to HSA near Subdomains IIA (where Trp 214 and Tyr 263 are located). Furthermore, the binding site of PpIX on HSA may be located near Subdomains IB (Tyr 138, Tyr 140, Tyr 148, Tyr 150, Tyr 161), IIB (Tyr 332, Tyr 334, Tyr 341) and/or IIIA (Tyr 401, Tyr 411, Tyr 452).

The spectroscopic studies were confirmed by the results of computer simulation. The docking results clearly indicate Subdomains IB (hemin binding site) and IIA (Sudlow’s Site 1) as the major binding sites for PpIX on the HSA molecule. Most likely, PpIX forms a strong “sandwich-type” complex between its highly conjugated porphine system and aromatic side chains of Trp and Tyr.

Finally, nine amino acid residues (Arg 117, Tyr 138, Ile 142, His 146, Phe 149, Phe 157, Tyr 161, Arg 186 and Lys 190) involved in the binding with PpIX in Site 1 of HSA are the same as those for heme, which was described by Wardell et al. [22]. This reveals that PpIX and heme may interact in Subdomain IIA of the tertiary structure of HAS.

## Figures and Tables

**Table 1:** Association constants (K_a_), number of binding sites (n) and Stern–Volmer constants (K_S-V_) for the PpIX-HSA complex.

	λ_ex_ = 280 nm	λ_ex_ = 295 nm
PpIX-HSA	K_a_ = 1.68 × 10^5^ M^−1^ *n* = 1	K_a_ = 2.30 × 10^5^ M^−1^ *n* = 1
	K_S-V_ = 1.78 × 10^5^ M^−1^	K_S-V_ = 1.93 × 10^5^ M^−1^

**Table 2:** Docking results for PpIX molecule inside HSA (1AO6.pdb) showing detailed analysis of the ligand binding sites, protein-ligand interaction energy and major residues involved in hydrogen bonding and hydrophobic interactions. Computational data obtained using the CLC DDWB program [23] and BIOVIA DS Visualizer software [24].

Binding Sites in HSA							PpIX-HSA Complexes		
Site	Volume (Å^3^)	Radius (Å)	Location	Domain	Subdomain	α-Helix	Energy (a.u.) ^a^	No. of H-bonds	Common Interacting Residues with PpIX
1	787.46	15	Core	I	B	h1, h2, h3, h4	−73.55	4 (6) ^b^	Arg 117, Tyr 138, Ile 142, His 146, Phe 149, Phe 157, Tyr 161, Arg 186
2	420.35	13	Core	I II III	B A B A	h4 h1, h2 h3 h3, h4	−70.34	3 (5) ^b^	Ala 191, Ala 194, Lys 195, Leu 198, Trp 214, Arg 218, Val 343, Lys 436, Lys 444, Pro 447, Cys 448, Asp 451, Tyr 452, Val 455
3	165.38	11	Surface	II III	A B A	h1, h2 h2, h3 h5, h6	−11.42	0 (2) ^b^	Phe 206, Arg 209, Ala 210, Lys 351
4	146.94	10	Surface	I	A B	h2, h3 h2, h3	−48.25	2 (4) ^b^	Glu 17, Ala 21, Ala 158, Lys 159, Lys 162
5	65.02	10	Surface	III	A B	h1, h2 h2, h3	−44.90	2 (4) ^b^	Leu 394, Leu 398, Ala 406, Arg 410, Lys 413, Thr 540, Lys 541
6	52.22	10	Surface	III	B	h1, h2	−33.56	1 (3) ^b^	Pro 421, His 510, Thr 506, Phe 509, His 510, Lys 524, Ala 528
7	52.22	10	Surface	III	A	h2, h5, h6	−49.05	2 (4) ^b^	Met 87, Gln 32, Gln 33, Lys 466

^a^ The unit of protein-ligand interaction energy is presented in arbitrary units (a.u.); the lower the value, the better the binding affinity; ^b^ including intramolecular H-bonds.

**Table 3:** Key protein-ligand contacts of bound PpIX to the ligand binding Sites 1 and 2 inside HSA (1AO6.pdb). Computational data obtained using the CLC DDWB program [23] and BIOVIA DS Visualizer software [24].

**Hydrogen Bonding Interactions**							
**Site 1**				**Site 2**			
PDB atom name	Ligand atom	Type of contact	Distance (Å)	PDB atom name	Ligand atom	Type of contact	Distance (Å)
(D) Arg117:HN (bb)	(A) PpIX:O(pp)	Conventional	2.02 *	(D) Lys444:HZ1 (sc)	(A) PpIX:O (pp)	Conventional	2.01 *
(D) Arg117:HE (sc)	(A) PpIX:O (pp)	Conventional	2.77 *	(D) Lys444:HZ1 (sc)	(A) PpIX:O (pp)	Conventional	2.25 *
(D) Tyr161:HH (sc)	(A) PpIX:O (pp)	Conventional	2.00 *	(D) Lys444:HZ2 (sc)	(A) PpIX:O (pp)	Conventional	2.58 *
(D) Arg186:HE (sc)	(A) PpIX: (r)	π-Donor Non classical	3.01 *		(D) PpIX:NH (r) (A) PpIX:N (r)	Conventional Intramolecular	2.21 *
	(D) PpIX:NH (r) (A) PpIX:N (r)	Conventional Intramolecular	2.21 *		(D) PpIX:NH (r) (A) PpIX:N (r)	Conventional Intramolecular	2.24 *
	(D) PpIX:NH (r) (A) PpIX:N (r)	Conventional Intramolecular	2.24 *				
**Hydrophobic Interactions**							
**Site 1**				**Site 2**			
PDB atom name	Ligand atom	Type of contact	Distance (Å)	PDB atom name	Ligand atom	Type of contact	Distance (Å)
Tyr138 (sc)	PpIX:C(v)	π-Alkyl	5.05	Ala191 (sc)	PpIX:C (v)	Alkyl	4.09
Ile142 (sc)	PpIX:C (m)	Alkyl	4.52	Ala191 (sc)	PpIX:C (m)	Alkyl	4.10
Ile142 (sc)	PpIX:C (v)	Alkyl	4.57	Ala194 (sc)	PpIX:C (v)	Alkyl	3.90
Ile142:CD1 (sc)	PpIX (pr)	π-σ	3.15	Lys195 (sc)	PpIX:C (v)	Alkyl	4.00
His146 (sc)	PpIX:C( m)	π-σ	3.62	Lys195 (sc)	PpIX (pr)	π-Alkyl	4.88
His146 (sc)	PpIX:C (v)	π-Alkyl	5.10	Leu198 (sc)	PpIX:C (m)	Alkyl	5.14
Phe149 (sc)	PpIX:C (m)	π-Alkyl	4.83	Trp214 (sc)	PpIX:C (v)	π-Alkyl	4.49
Phe157 (sc)	PpIX:C (v)	π-Alkyl	4.50	Trp214 (sc)	PpIX:C (v)	π-Alkyl	4.61
Arg186 (sc)	PpIX (chms)	π-Alkyl	3.11	Arg218 (sc)	PpIX:C (v)	Alkyl	4.55
Arg186 (sc)	PpIX (pr)	π-Alkyl	3.46	Val343 (sc)	PpIX:C (v)	Alkyl	3.49
Arg186 (sc)	PpIX (pr)	π-Alkyl	4.35	Lys436 (sc)	PpIX:C (m)	Alkyl	5.31
Arg186 (sc)	PpIX (pr)	π-Alkyl	4.72	Lys436 (sc)	PpIX:C (m)	Alkyl	3.79
Arg186 (sc)	PpIX (pr)	π-Alkyl	5.42	Lys436 (sc)	PpIX (pr)	π-Alkyl	5.27
				Pro447 (sc)	PpIX (pr)	π-Alkyl	5.21
Lys190 (sc)	PpIX:C (v)	Alkyl	4.43	Pro447:C,O (bb) Cys448:N (bb)	PpIX (pr)	Amide π-Stacked	3.94
Lys190 (sc)	PpIX:C (m)	Alkyl	4.72	Cys448 (sc)	PpIX (pr)	π-Alkyl	5.32
Lys190:CD1 (sc)	PpIX (pr)	π-σ	3.94	Cys448 (sc)	PpIX (chms)	π-Alkyl	5.33
Lys190 (sc)	PpIX (pr)	π-Alkyl	5.27	Asp451:C,O (bb) Tyr452:N (bb)	PpIX (pr)	Amide π-Stacked	4.47
				Asp451:C,O (bb) Tyr452:N (bb)	PpIX (chms)	Amide π-Stacked	5.66
				Tyr452 (sc)	PpIX (pr)	π-π T-shaped	4.96
				Tyr452 (sc)	PpIX (m)	π-Alkyl	4.59
				Tyr452 (sc)	PpIX (v)	π-Alkyl	5.48
				Val455 (sc)	PpIX:C (m)	Alkyl	4.56
				Val455 (sc)	PpIX:C (v)	Alkyl	3.97
				Val455 (sc)	PpIX (pr)	π-Alkyl	5.43
**Miscellaneous Interactions**				**Unfavorable Interactions**			
**Site 2**				**Site 2**			
PDB atom name	Ligand atom	Type of contact	Distance (Å)	PDB atom name	Ligand atom	Type of contact	Distance (Å)
Cys448:SG (sc)	PpIX (pr)	π-Sulfur	3.76	Lys436:HZ2 (sc)	PpIX:C (m)	Steric Bumps	1.04
				Lys436:HZ3 (sc)	PpIX:H (m)	Steric Bumps	1.34
				Lys436:NZ (sc)	PpIX:H (m)	Steric Bumps	1.37
				Lys436:HZ2 (sc)	PpIX:C (m)	Steric Bumps	1.64
				Lys436:NZ (sc)	PpIX:C (m)	Steric Bumps	2.20

* Strong H-bond (max distance = 3.4 Å), weak H-bond (max distance = 3.8 Å); (A), H-bond acceptor; (D), H-bond donor; (sc), side chain functional group of the amino acid; (bb), backbone; (pp), propionic acid group; (v), vinyl group; (m), methyl group; (pr), pyrrole ring; (chms), conjugated heterocyclic macrocycle system.
